# Commentary: The magnetic touch illusion: A perceptual correlate of visuo-tactile integration in peripersonal space

**DOI:** 10.3389/fnhum.2016.00492

**Published:** 2016-10-13

**Authors:** Francesca Ferri, Marcello Costantini

**Affiliations:** Department of Psychology, Centre for Brain Science, University of EssexColchester, UK

**Keywords:** rubber hand illusion, multisensory integration, expectation, bodily illusion, bodily self-consciousness

An intriguing paper by Guterstam et al. (2016) has recently shown a new perceptual illusion, namely the “magnetic touch illusion.” It is elicited by synchronous brushstrokes applied to a participant's hidden real hand and brushstrokes delivered at some distance above the rubber hand, but never actually touching it.

The main features of the magnetic touch illusion are: (i) the non-linear decay at distances >40 cm from the rubber hand and its being spatially anchored to the position of the rubber hand; (ii) the dependency upon actual multisensory information. Indeed, mere tactile expectation is not sufficient to induce it; (iii) the necessity of a continuum spatial representation between the brush in mid-air and the rubber hand. The illusion could not be induced when a physical barrier is introduced between them.

In this comment we focus on the second feature of the magnetic touch illusion and the proposal that tactile expectation is not sufficient to induce it. In Guterstam's study two out of eight experiments (2A and 2B) were motivated by our previous studies (Ferri et al., 2013, 2014) showing that expectation of tactile stimulation alone, in the form of an object slowly approaching the rubber hand without touching it, is sufficient to induce a vivid illusion of rubber hand ownership.

In our study, participants were seated with their right arm resting upon a table just below another smaller table. Hence, the real hand was hidden from the participant's view and a life-sized rubber model of a right hand was placed on the small table, vertically aligned with the participant's hidden hand. Participants observed the experimenter's hand while approaching—without touching—the rubber hand. We quantified the phenomenology of the illusion by means of explicit (i.e., subjective report in the form of questionnaires) and implicit (i.e., SCR, Skin Conductance Response) measures. Both measures indicated that participants experienced the illusion that the experimenter's hand was about to touch their hidden hand rather than the rubber hand, as if the latter had replaced their own hand. Implicit responses further qualified subjective measures showing that embodiment of the rubber hand occurred once the approaching hand entered participants' peripersonal space (< 45 cm).

According to Guterstam et al. (2016) the discrepancy between (Experiments 2A and 2B, visual only—approaching brush condition) and our findings might be easily explained by cognitive bias, suggestibility and task compliance in our participants. In this comment we reason that these alternative interpretations can unlikely explain our findings, for at least two reasons. First, while cognitive bias, suggestibility, and task compliance could easily contaminate subjective reports, it is highly unlikely that they contaminate implicit responses in any ways. Second, as we found an increase in skin conductance responses only when the approaching object entered participants' peripersonal space, cognitive bias, suggestibility and task compliance would contaminate implicit responses in a spatially selective manner, which is very hard to conceive.

In our view, a possible explanation for the contrasting results could be that while Guterstam et al. (2016) successfully induced tactile expectation on the rubber hand, they failed to induce any tactile expectations on the real hand. As in the case of the standard rubber hand illusion, where synchronous visuo-tactile stimulation needs to be delivered on both hands (Botvinick and Cohen, 1998; Ehrsson, 2012; Costantini, 2014), tactile expectation should be generated on both hands to induce a vivid illusion of rubber hand ownership.

To build expectation about forthcoming events our brain exploits prior experiences of spatial and temporal regularities characterizing causal relationship between objects in the environment. For example, in a mechanical event with two objects, spatial continuity and temporal contiguity increase the likelihood that a person will perceive causality (e.g., Straube and Chatterjee, 2010; Woods et al., 2014). When one object—for instance, a billiard ball—moves toward another, the path and timing of movement of both objects influence our expectation of whether one object will cause the other to move. In the case of the magnetic touch illusion, expectation cannot be induced because the approaching object follow a path that is compatible with a collision with the rubber hand, but incompatible with a collision with the real hand. In Guterstam et al. (2016) study (Experiments 2A and 2B), indeed, the rubber and the participant's hands, were not vertically aligned, as they were displaced by 15 cm on the horizontal plane. According to us, such spatial misalignment prevented the formation of tactile expectation, as the approaching object moving along a path toward the rubber hand, would never, even potentially, touch the real hand. Hence, in Guterstam et al. (2016) study neither an actual tactile stimulus is delivered on the real hand nor tactile expectation is generated on the same hand.

The relevance of movement path in attending approaching objects has been demonstrated also in other domains. For instance, Lin et al. (2008) asked participants to perform a visual search task of approaching stimuli on a collision path, or on a near-miss collision path with the observer. Results showed more accurate and faster responses to target moving on a collision path with the observer than when it was moving on a near-miss path.

Based on this evidence, we suggest that in Guterstam's study participants did not experience the illusion not because expectation is not sufficient to induce it, rather because they did not allow the formation of any expectations of touch on the real hand. We also suggest that the procedure employed by Guterstam et al. (2016) could be effective in eliciting the magnetic touch illusion once the ownership illusion has been previously induced with a standard synchronous visuo-tactile stimulation. This is because, once illusory ownership is induced, visual receptive field of visuo-tactile neurons shift toward the rubber hand (Graziano et al., 2000), making the necessary condition of vertically aligned hand irrelevant.

Future studies should investigate the effect of spatial alignment between the approaching object the real and rubber hands on the magnetic touch illusion. Our prediction is that tactile expectation is sufficient to induce the magnetic touch illusion provided that the path of the approaching object is compatible with a collision with both the real and the rubber hands.

## Author contributions

FF and MC wrote and approved the commentary.

### Conflict of interest statement

The authors declare that the research was conducted in the absence of any commercial or financial relationships that could be construed as a potential conflict of interest.
